# Marked variability in bioactivity between commercially available bovine colostrum for human use; implications for clinical trials

**DOI:** 10.1371/journal.pone.0234719

**Published:** 2020-06-17

**Authors:** Raymond J. Playford, Meg Cattell, Tania Marchbank

**Affiliations:** 1 Centre for Immunobiology, Blizard Institute, Barts and The London School of Medicine, Queen Mary, University of London, London, England, United Kingdom; 2 Peninsula Medical School, University of Plymouth, Plymouth, England, United Kingdom; 3 Department of Research & Development, Pantheryx Inc, Boulder, Colorado, United States of America; Keele University School of Medicine, UNITED KINGDOM

## Abstract

**Background:**

Colostrum, the milk produced during first few days after birth, is rich in immunoglobulins, antimicrobial peptides & growth factors. Multiple clinical trials using bovine colostrum are ongoing but with no assessment of test product bioactivity.

**Objectives:**

To examine variability of bioactivity between 20 commercial colostrum products, contribution of TGFβ and EGFR in mediating effects, heat sensitivity of bioactivity and changes in bioactivity of colostrum milkings in the days following calving.

**Design:**

In vitro bioactivity used AGS, RIE-1 and Caco-2 cell proliferation (Alamar blue) and migration (wounded monolayers) assays. Changes in colostrum bioactivity determined following addition of TGFβ-neutralising antibody, EGFR blocker (Typhostin) and after heating (40–60°C, 60 min). In vivo bioassay assessed ability of colostrum gavage (2ml, 7mg/ml) to reduce gastric damage (NSAID + restraint) in rats. Milkings from 6 cows, days 0–3 post calving were assessed for bioactivity and growth factor concentrations.

**Result:**

Six-fold differences in pro-proliferative and migratory activity were seen comparing commercial products. Comparison of most- and least-active samples from in vitro studies showed two- to three-fold differences in ability to reduce gastric injury (86% reduction using most-active vs 48% using least-active, p<0.01). Tyrphostin reduced pro-migratory and proliferative activity by 23% and 55%. TGFβ neutralisation reduced migratory activity by 83% but did not affect proliferation Heating colostrum powder to 50°C did not affect immunoactivity of haptoglobin, EGF, TGFβ, IgG, IGF-1 or betacellulin but decreased bioactivity by >40%. Milking studies showed high bioactivity during first and second milkings on day 0 but 77% reduction by day 3. Changes in total protein, haptoglobin, EGF, TGFβ, IgG and IGF-1 paralleled falls in bioactivity.

**Conclusion:**

Commercial colostrum products possess widely different bioactivity. Variation in heat exposure and/or proportion of day 0 colostrum content may contribute to this. Assessment of colostrum bioactivity has advantages to growth factor quantitation for quality control.

## Introduction

Colostrum is the specific first diet of mammalian neonates and is rich in immunoglobulins, antimicrobial peptides e.g. lactoferrin, and other bioactive molecules including growth factors such as transforming growth factor-beta (TGFβ) and insulin-like growth factor-1 (IGF-1) [1]. In combination with the milk that is subsequently produced, it is important for the nutrition, growth, and development of the new-born infant mammal. It also contributes to the immunological defence of the neonate and in eliminating infection and stimulating growth of the neonatal gastrointestinal tract [2].

Bovine (and human) colostrum contain over twenty different molecules with growth factor/pro-reparative activities [1, 3]. Although the relative concentration of individual growth factors varies between species [4], colostrum from cows and camels have been shown to stimulate growth of human cell lines, confirming cross species bioactivity [1, 5]. Bovine colostrum is a side product of the dairy industry and is easily accessible in bulk, relatively low cost to produce and, in contrast to the use of human colostrum, acceptable to most consumers. It is for these reasons that most studies examining the value of colostrum for adult gut health issues use bovine, rather than human colostrum.

In adults, randomised clinical trials relating to gut damage have shown beneficial effects of oral bovine colostrum supplementation in reducing NSAID and exercise induced hyperpermeability [6, 7] and, when given by enema, to enhance the recovery of ulcerative colitis [8]. There is also preliminary data on the potential use of colostrum for a diverse range of pathologies including prevention of upper respiratory tract infections and preservation of muscle mass [9, 10]. There are currently over 30 clinical trials underway worldwide including in the US, UK and African continents studying the effect of bovine colostrum in both neonates for conditions such as necrotising enterocolitis and neonatal enteropathy/stunting and in adults for conditions such as chemotherapy induced mucositis and treatment of critically ill patients (e.g. https://trialbulletin.com/lib/trials/term=Colostrum).

Most adult clinical trials examining the use of colostrum for gut disorders used a dose of between 20-40g colostrum powder/day. However, as highlighted by Rathe in his review of clinical trials involving colostrum [11], optimal dosage and duration of bovine colostrum supplementation have not been established. It also highlights a serious confounding factor in that investigators base dosage on the weight of the dry powder of colostrum rather than establishing equivalent bioactivity, which may be influenced by breed, herd, milking times, and formulation This leads Rathe to conclude that simple expressions of dosage in weight units are not sufficiently informative and some form of bioactivity standardization needs to be undertaken [11].

Quality control assessments of commercial colostrum are usually restricted to quoting total protein concentration and immunoglobulin levels, neither of which give any indication as to pro-reparative activity. There are several potential concerns relating to consistency of colostrum both within and between clinical studies; 1. Producers usually ship product and recommend storage at room temperature (Table 1). However, many studies are conducted in countries or environments where high ambient temperature occur which may influence bioactivity. 2. Commercial colostrum products do not specify either individual growth factor concentrations or, of more value, total “growth factor” bioactivity. 3. There is no strict definition of how many days following calving the product can be considered as colostrum rather than milk, although it is generally accepted colostrum can only be considered as such up to day 3 following calving [3]. Differences in bioactivity between producers may, therefore, exist if the proportion of colostrum derived from different days post calving varies between suppliers.

**Table 1:** Commercial colostrum sample information provided by producer.

COLOSTRUM	Total stated protein (g/100 g)	Form	Source	Total IgG (g/100g)	Recommended storage	Lot number
Neovite lactose reduced first milk	70	Powder	UK	28	Cool dry place	1805082
Neovite whole colostrum from Welsh farms	55	Powder	UK	16.5	Cool dry place	SA44-01
Neovite cow’s first milk	55	Powder	UK	16.5	Cool dry place	1801007
Colodan whole colostrum	NS	Powder	Denmark	13	Cool dry place	B5032-017
Bulkpowders colostrum	63.3	Powder	Germany	18.99	Cool dry place	NS
Biestmilch	70	Capsule	Hawaii	NS	Store for up to 3 year at RT	171241
Vitacost colostrum ultra	NS	Capsule	USA	40	Room Temp 15–30°C	3823400
Douglas Labs colostrum	NS	Powder	New Zealand	NS	Cool dry place 15–25°C	0153284
Immune Tree colostrum	66.7	Powder	USA	NS	Cool dry place	9902/143
Nutracost	NS	Capsule	USA	NS	NS (shipped at RT)	18010466
NOW colostrum powder	NS	Powder	USA	NS	Cool dry place	3046338
Nutrablast	NS	Capsule	USA	7	Cool dry place	279331
Sovereign Labs colostrum	60	Powder	USA	5	Cool dry place	1802027
Synertek Intact Balanced First colostrum	66.7	Powder	USA	NS	Cool dry place <25°C	657–30
Renegade Pharmacist	66.7	Powder	USA	22.11	NS (shipped at RT)	9902/221
Sterling colostrum 2070	70	Powder	USA	NS	NS (shipped at RT)	023741
TBR labs peptide ignition colostrum	66.7	Powder	USA	13	Cool dry place	03028219
Sterling colostrum 3070	70	Powder	USA	25	NS (shipped at RT)	2396–9
Glanbia high fat WPC	88	Powder	USA	NS	NS (shipped at RT)	0068701
Standard colostrum	70	Powder	USA	30	Store at RT for up to 3 years	1141–048203 4518Fi

Table shows source of colostrum samples and product data sheet information for total protein, total IgG, lot numbers and recommended storage conditions, NS = not stated. RT = (store at) room temperature. Storage advice was either present on data sheet or through direct contact with producer. NB list of products are described in random order and do not relate to the order of bioactivity.

To address these concerns relating to quality control, we therefore performed a series of interrelated studies: 1) To determine the amount of variability of twenty commercial colostrum samples obtained worldwide in their ability to stimulate proliferation and migration of intestinal cells lines. 2) Having found major variation exists, we studied the most and least active colostrum samples using an in vivo model of gastric damage to examine if differences seen in vitro had pathophysiological-therapeutic relevance 3) We then studied an exemplar colostrum sample in more detail to determine the importance of the EGFR and TGFβ pathways in mediating its proliferative and migratory effects and whether exposing the colostrum powder to heat (as could occur during transportation or storage) affected in vitro proliferation and migration activity and/or the immunoreactivity of various growth factors. 5) Finally, we examined whether the date of collection of colostrum post calving had a major influence on bioactivity and/or concentrations of a variety of growth factors present in the colostrum.

## Materials and methods

### Ethics

All animal experiments were approved by the Local Animals Ethics Committee (Queen Mary’s University of London Animal Welfare Committee) and covered by project (PO13B304A) and personal (IE9346EEF) license under the Home Office Animals Procedures Acts, 1986.

### Cell lines

Caco-2 is derived from a colorectal adenocarcinoma of 72-year-old male (ATCC® HTB37^TM^, ATCC, LGC standards, Teddington, UK) and exhibits tight junctions and desmosomes between adjacent cells and grows as polarized monolayers [12]. AGS is derived from gastric adenocarcinoma of a 54-year-old female (ATCC® CRL-1739™, ATCC, LGC standards, Teddington, UK) [13], RIE-1 is a spontaneously immortalized rat intestinal epithelial cell line (gift from K Brown, Babraham Institute, Cambridge, UK) [14]. Cells were grown in Dulbecco’s modified Eagle medium ((DMEM) Caco-2 and RIE-1) or RPMI 1640 medium (AGS) containing 10% fetal calf serum at 37°C in 5% CO2. All cells are routinely mycoplasma tested.

## Study series 1

### A. Variation in in vitro bioactivity of commercial products

#### Background to protocol

These studies examined variation in bioactivity between different commercial samples using in vitro models of the early stages of gut repair i.e. cell migration and proliferation.

#### Commercial colostrum samples

20 commercial bovine colostrum samples promoted as health food supplements were purchased via the internet. Source countries of these samples included mainland USA, Hawaii, Denmark, Germany, UK and New Zealand. All products stated that they are 100% colostrum (Table 1).

#### Preparation of samples

Samples that were already in powdered form or collected from capsules were dissolved in phosphate buffered saline (PBS, Sigma) to a concentration of 10 mg/ml with vortexing. Subsequent dilutions were made in serum free medium (SFM) for cell migration and cell proliferation assays. For immunoassays, samples were diluted in relevant assay buffer. For in vivo experiments, colostrum samples for gavage were dissolved in sterile water. Protein assays were performed using a standard Bicinchoninic Acid (BCA) assay (Sigma, Poole, Dorset, UK).

#### Proliferation assay methods

Cell proliferation assays were performed as previously described [15], utilising Alamar blue (Invitrogen, Paisley, UK), as per manufacturer’s instructions. Briefly, AGS cells, Caco-2 cells, or RIE-1 cells were seeded at 2000 cells/well, grown in medium and 10% FCS in 96 well plates overnight (20 hours). Cells then had the medium removed and were washed twice using 200 μl of SFM using a multichannel pipette (Anachem, Beaumont Leys, Leicester, UK) and incubated in colostrum preparations, or SFM alone as a negative control. In addition, as in our previous studies using this method [16], additional wells had a standard dose of EGF (1 μg/ml in SFM) added as a positive control where the rise in proliferation (increase in A570 above baseline) caused by the EGF is defined as 100%. This provides the potential to compare relative effects across different assay days where the absolute values will vary. All results are expressed as mean +/- SEM for quadruplicate wells.

Pilot studies using (0.5–4 mg powder/ml) determined the optimum concentration for subsequent in vitro experiments to be 1 mg powder/ml. The dose-response curve showed that higher concentrations did not stimulate proliferation further and at a concentration of 4 mg/ml, values decreased due to the toxic effect of the colostrum causing the cells to become detached (S1 Fig).

All 20 commercial colostrum products were assessed for pro-proliferative activity in the three cell lines using 1 mg powder weight/ml. In addition, we compared all 20 samples in their ability to stimulate proliferation of AGS cells when the amount of colostrum sample added was standardised according to its protein content such that each well received 0.6 mg total colostrum protein/ml (rather than 1 mg/ml of colostrum powder weight).

#### Cell migration assay methods

Cell migration assays were performed using AGS, Caco-2 and RIE-1 cells using our previously published methods [17]. Briefly, confluent monolayers were grown in 24 well plates in 10% serum containing medium. Standard wounds were inflicted by scraping a pipette tip across the monolayer and medium removed. Cells were washed immediately with 2 ml of serum-free medium to remove released soluble factors. Cells were then incubated in SFM alone (negative control), or SFM containing the colostrum samples. In addition, as in our previous studies [16] further wells had EGF (1 μg/ml in SFM) added as a positive control where the amount of movement caused by EGF was defined as 100%, providing the potential to compare relative effects across different assay days where the absolute values will vary.

Serial photomicrographs were taken using an inverted microscope (Nikon TS100; Tokyo, Japan) and a Nikon Coolpix 800 digital camera with 125-fold magnification [18]. Twenty measurements per field were performed by placing a transparent grid over the photograph and measuring the distance moved from the original wound line. The exact width of the wound is therefore not relevant as it is the movement of the wound edge from time zero that is assessed.

As for the proliferation assays, all 20 commercial colostrum products were assessed for pro-migratory activity in all three cell lines using 1 mg powder weight/ml and additional studies performed comparing all 20 samples in their ability to stimulate migration of AGS cells when the amount of colostrum sample added was standardised according to its protein content such that each well received 0.6 mg total colostrum protein/ml (rather than 1 mg/ml of colostrum powder weight). All results are expressed as mean +/- SEM for triplicate wells.

### B. Pathophysiological relevance of in vitro differences in bioactivity

#### Background to protocol

**Fig 1:**
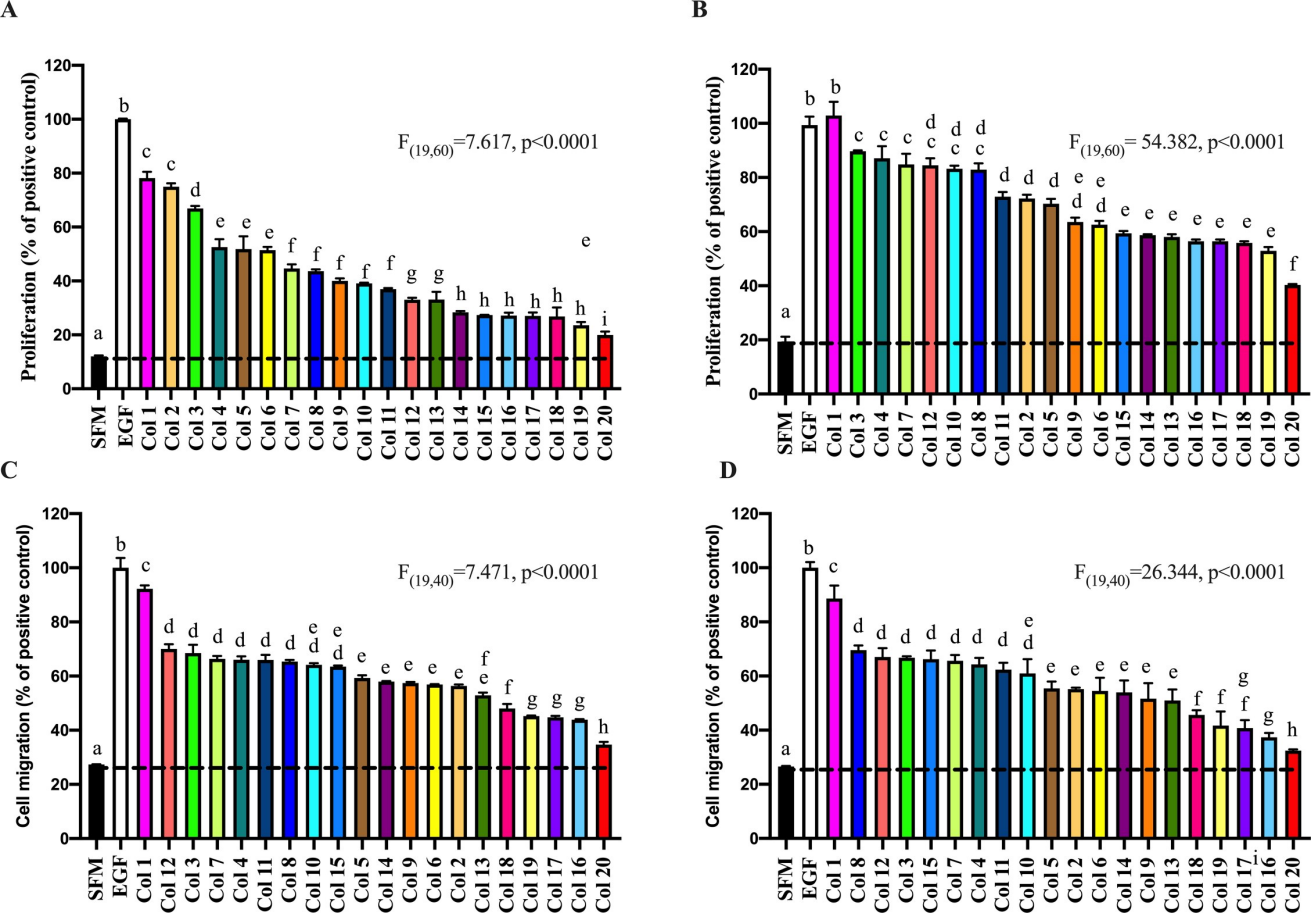
Variation in pro-proliferative and pro-migratory bioactivity of commercial colostrum samples.

To examine whether differences in in vitro bioactivity found in study 1A had pathophysiological relevance, we used a rat gastric damage model to compare the samples with the most- and least-stimulatory bioactivity (colostrum sample 1 or 20 from Fig 1A) in their ability to reduce gastric damage.

#### Rat gastric damaging model

The ability of colostrum to prevent gastric damage in rats stressed by indomethacin and restraint was assessed using our previously validated model [6]. Gastric damage is induced through the combination of the toxic NSAID effects of the indomethacin which is enhanced by the short-term (3 hour) stress of restraint [19]. The indomethacin is not given to provide analgesia and it is of note that opiate analgesia cannot be given as it reduces acid secretion preventing injury occurring [19].

Briefly, Sprague Dawley rats (all males, N = 8 per group, 200–225 g) received 2 ml total gavage volume containing either the most-active or least-active performing colostrum from the in vitro studies (sample 1 or sample 20 from Fig 1A) at 7 mg/ml, wt/vol. This dose of colostrum was chosen based on our previous studies examining effects of a colostrum product in this model [6]. An additional group received the same gavage volume containing BSA (7 mg/ml) as a negative control. Mean body weight for each of the 3 groups were control 215.1 +/-, 4.4 g animals receiving most active colostrum 215.4 +/- 3.5 g, animals receiving least active colostrum 215.0 +/- 2.1 g, no significant difference between groups.

All gavage solutions also contained 2% hydroxymethylpropylcellulose (Sigma) to reduce the rate of gastric emptying. Thirty minutes after gavage, all rats received indomethacin (20 mg/kg, subcutaneously, Sigma) and were placed in Bollman type restraint cages. Animals were killed three hours later using a Schedule 1 method (inhalation of carbon dioxide gas) and their stomachs removed and inflated with 4 ml of 10% formalin (Sigma). The stomachs were randomly coded and macroscopic and microscopic assessment of injury assessed in a blinded fashion. Macroscopic injury was assessed using a dissecting microscope (×10) with the aid of a reference square grid and reported as the total area of ulceration per stomach (mm^2^/stomach). The stomachs were then embedded in wax and the depth of damage assessed microscopically as previously described [6]. Briefly, microscopic injury was graded with a score from 0 to 4 where: 0 = no damage,1 = one small erosion (>0.5 mm), 2 = two small or one large erosion (< 0.5 mm), 3 = two or more large erosions, and 4 = any area of ulceration extending to the muscularis mucosa.

### C. Relevance of EGFR, TGFβ and heat stability on proliferative and migratory activity

#### Background to studies

We examined the importance of TGFβ and EGFR pathways in mediating in vitro bioactivity and the effect of short-term heat exposure on bioactivity and immunoreactivity. Given the large number of measurements to be undertaken, we identified a typical colostrum sample from study Series 1 (sample 9), which ranked at the midpoint of the relative bioactivity of the 20 samples for these more detailed analyses.

#### Immunoneutralization studies

Colostrum sample 9 was added to wells at 1 mg powder/ml in the presence and absence of an EGFR blocker (Tyrphostin, 100 nM, Sigma) to examine the EGFR pathway or a TGFβ (100μg/ml) neutralising antibody (antibodies-online.com, Aachen, Germany) to examine the importance of the TGFβ pathway and assessed for pro-proliferative and migratory activity.

#### Heat stability studies

Four 5g aliquots of dry powder colostrum sample 9 were placed into falcon tubes. Three of these were heated for 1 h at 40, 50 or 60°C by placing in a laboratory oven under continuous rotation and the fourth kept at laboratory room temperature (21°C, control) throughout. Samples were removed from oven and left overnight to return to room temperature. Samples were subsequently diluted to 1 mg powder/ml and analysed using proliferation and migration assays and for growth factor immunoactivity. To show that protein concentration had not been altered by the heating, protein concentration of samples was determined before and after using BCA assay.

## Study series 2

### Variation in bioactivity and growth factor immunoactivity in colostrum samples during days 0–3 after calving

#### Collection, processing, and assays of milkings

Colostrum samples were collected from 6 multiparous Holstein cows during January 2019. Cows were sampled at first and second colostrum milking and daily for the following 3 days. Colostrum was harvested into individual milking buckets, agitated, and subsampled with a stainless-steel dipper into 20 mL plastic vials and frozen within 30 min after collection. Samples were kept frozen at -20°C until freeze drying by APS Biogroup (Phoenix, AZ). All animals provided specimens for each time point.

Samples were subsequently analysed for pro-proliferative and pro-migratory effects and levels of IgG and growth factors determined using commercial ELISA kits according to manufacturer’s instructions. Measured components comprised bovine EGF, bovine TGFβ, bovine haptoglobin, and bovine betacellulin (from antibodies-online.com, Aachen, Germany), bovine IgG and IGF-1 from R&D systems (Abingdon, UK) and bovine betacellulin from Abcam (Cambridge, UK).

## Statistics

All results are expressed as mean ± SEM. Statistics were performed using Graphpad Prism 8 version 8.3.1. Test for normality of data using Shapiro Wilks test showed equal variances between groups. Commercial samples studies were assessed using one-way analysis of variance (ANOVA). Comparisons between treatments was performed using a Tukey’s multiple comparison test. For cell migration and proliferation assays, N = 3 or 4 wells per treatment were used to generate a power of 0.76 and 0.8, respectively, for an effect of change of 50% and a significance of p<0.05. For rat gastric damaging model, N = 8 per group were used to generate a power of 0.83 for an effect of change in damage of 25% and a significance of p<0.05. For study series 2, a 2-way ANOVA was used with cow and time as factors. Where a significant effect was seen (p<0.05) in the ANOVAs, individual comparisons between groups were performed based on the group means, residual, and degrees of freedom obtained from the ANOVA, a method equivalent to repeated measures analysis.

## Results

### Study series 1

#### A. Variation in bioactivity of commercial products

A wide range of pro-proliferative (Fig 1A) and pro-migratory (Fig 1C) activity was seen, with an approximate 6-fold difference between the most active and least active samples when tested on a mg powder per ml basis. Relative bioactivity across the two assays were generally consistent with the most active and least active samples being similar in both assays. Similar results were seen when samples were tested using a standardised mg colostrum protein/ml basis (Fig 1B and 1D) rather than a standardised colostrum powder weight/ml basis.

AGS cells were incubated in 1 mg powder/ml (A&C) or a standardised amount of total colostrum protein, 0.6 mg protein/ml (B&D) of 20 different commercial colostrum samples for 24h. Changes in proliferation assessed using (Alamar Blue) (A&B) and movement of leading edge of wounded monolayers (C&D) determined. Results expressed as % response compared to effect caused by adding 1μg/ml EGF (positive control, defined as 100%). Colostrum sample numbers and colouring in A-D remain consistent taken from Fig 1A. SFM shows result of serum free medium alone (baseline control). Results expressed as means +/- SEM of 4 wells (proliferation assays) or 3 wells (migration assays). Results of one-way ANOVA showed significant differences between colostrum samples. Labelled means without a common letter are significantly different, P<0.05. Similar results seen using Caco-2 or RIE-1 cells (S2 Fig and S3 Fig).

#### B. Pathophysiological relevance of in vitro differences in bioactivity

**Fig 2:**
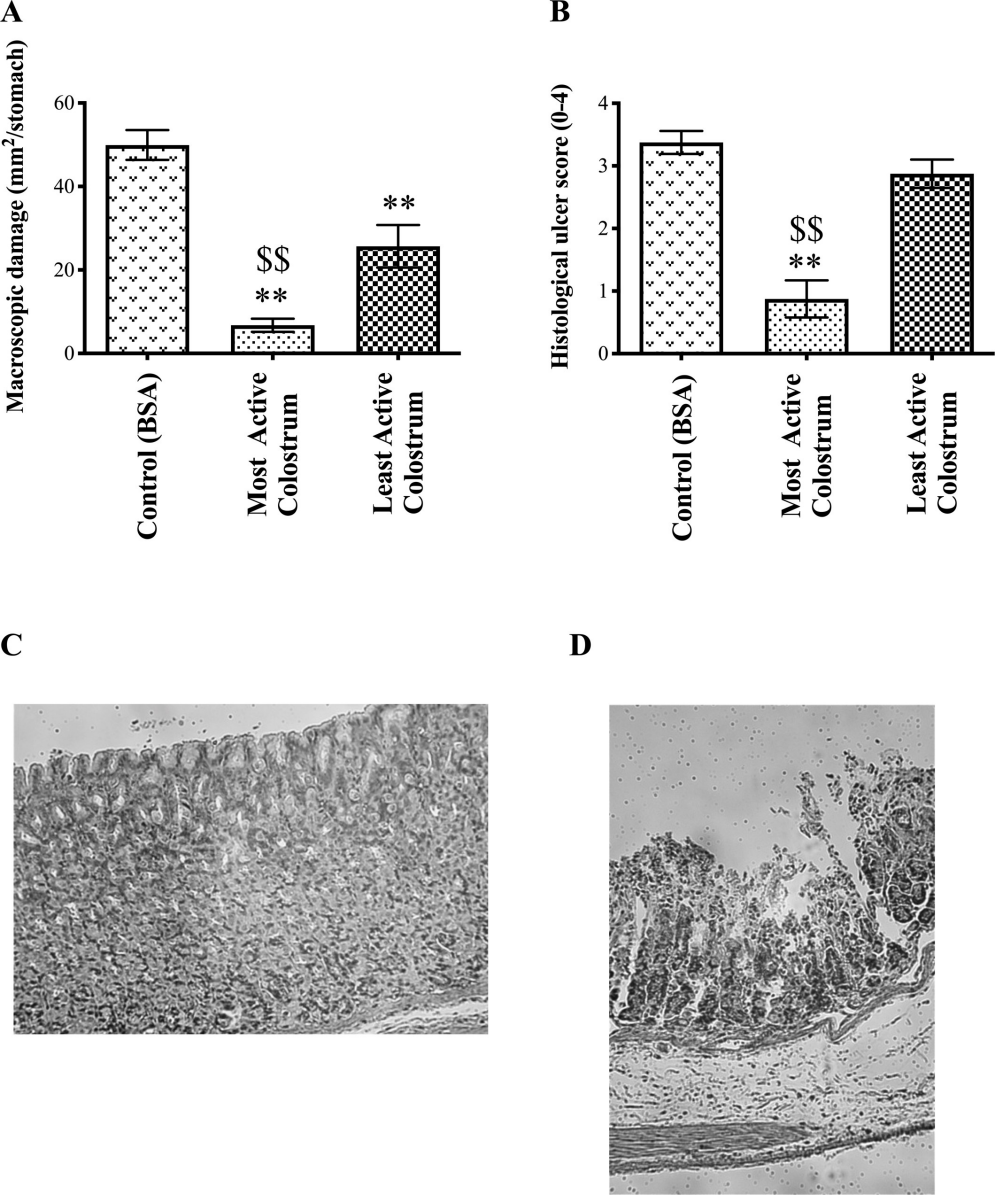
Effect of colostrum samples on NSAID-induced gastric injury. Rats received 2ml oral gavage of control (BSA), or the most active or least active colostrum samples determined from Fig 1A (colostrum samples numbered 1 and 20), 30 minutes prior to receiving indomethacin (20mg/kg, sc), and 3h of restraint. Amount of macroscopic (A) and microscopic (B) damage was subsequently assessed. Microscopic scoring scheme described in [3]. Stomachs from animals that received NSAID + most active colostrum had essentially normal histology (C) whereas animals receiving NSAID + least active colostrum continued to show multiple erosions (D). Results expressed as mean +/- SEM of 8 animals per group. ** signifies p< 0.01 vs control and $ $ signifies p< 0.01 of best sample vs worst.

All animals completed the study protocol. Most active (sample 1) and least active (sample 20) colostrum samples showed about a two-fold difference in the degree of reduction in macroscopic injury (86% reduction in macroscopic injury using most active sample vs 48% using least active, p<0.01, Fig 2A). Similar results were seen using microscopic scoring with a three- to four-fold difference in protective ability (74% reduction in injury using most active vs 15% using least active sample, p<0.01, Fig 2B). Animals receiving most active colostrum showed virtually normal gastric histology (Fig 2C), whereas animals receiving least active colostrum continued to have multiple erosions present (Fig 2D).

#### C. Relevance of EGFR, TGFβ and heat stability on proliferative and migratory activity

*Immunoneutralization studies*. Pro-proliferative activity of the colostrum sample 9 was reduced by 55% in the presence of the EGFR blocker Tyrphostin (p<0.01). The addition of TGFβ neutralising antibody did not affect the pro-proliferative effect of the colostrum (Table 2).

**Table 2:** Importance of EGFR and TGFβ on proliferative and migratory activity of colostrum.

	SFM alone	Colostrum alone	Colostrum + EGFR blocker (Tyrphostin)	Colostrum + TGFβ neutralising antibody
Proliferation (% of positive control, EGF 1 μg/ml = 100%)	43.18 +/- 5.1 **	123.3 +/- 8.5	78.7 +/- 2.4**	118.2 +/- 5.5 (NS, p = 0.214))
Cell migration (% of positive control, EGF 1 μg/ml = 100%)	27.2 +/- 2.8 **	69.3 +/- 0.2	59.6 +/- 1.3**	34.9 +/- 0.2**

AGS cells were incubated in 1 mg powder/ml colostrum +/- EGFR blocker (Tyrphostin, 100 nM) or a TGFβ (100 μg/ml) neutralising antibody for 24h. Changes in proliferation assessed using (Alamar Blue) and movement of leading edge of wounded monolayers determined. Results expressed as % response compared to effect caused by adding 1μg/ml EGF (positive control, defined as 100%). SFM shows result of serum free medium alone (baseline control). Results expressed as means +/- SEM of 4 wells (proliferation assays) or 3 wells (migration assays).

** signifies p<0.01 vs colostrum alone. NS- no significant difference vs colostrum alone

Pro-migratory activity of the colostrum sample was reduced by 23% in the presence of the EGFR blocker Tyrophostin (p<0.01). Similarly, the addition of TGFβ neutralising antibody caused pro-migratory activity of colostrum to fall by 83% (p<0.01, Table 2).

**Fig 3:**
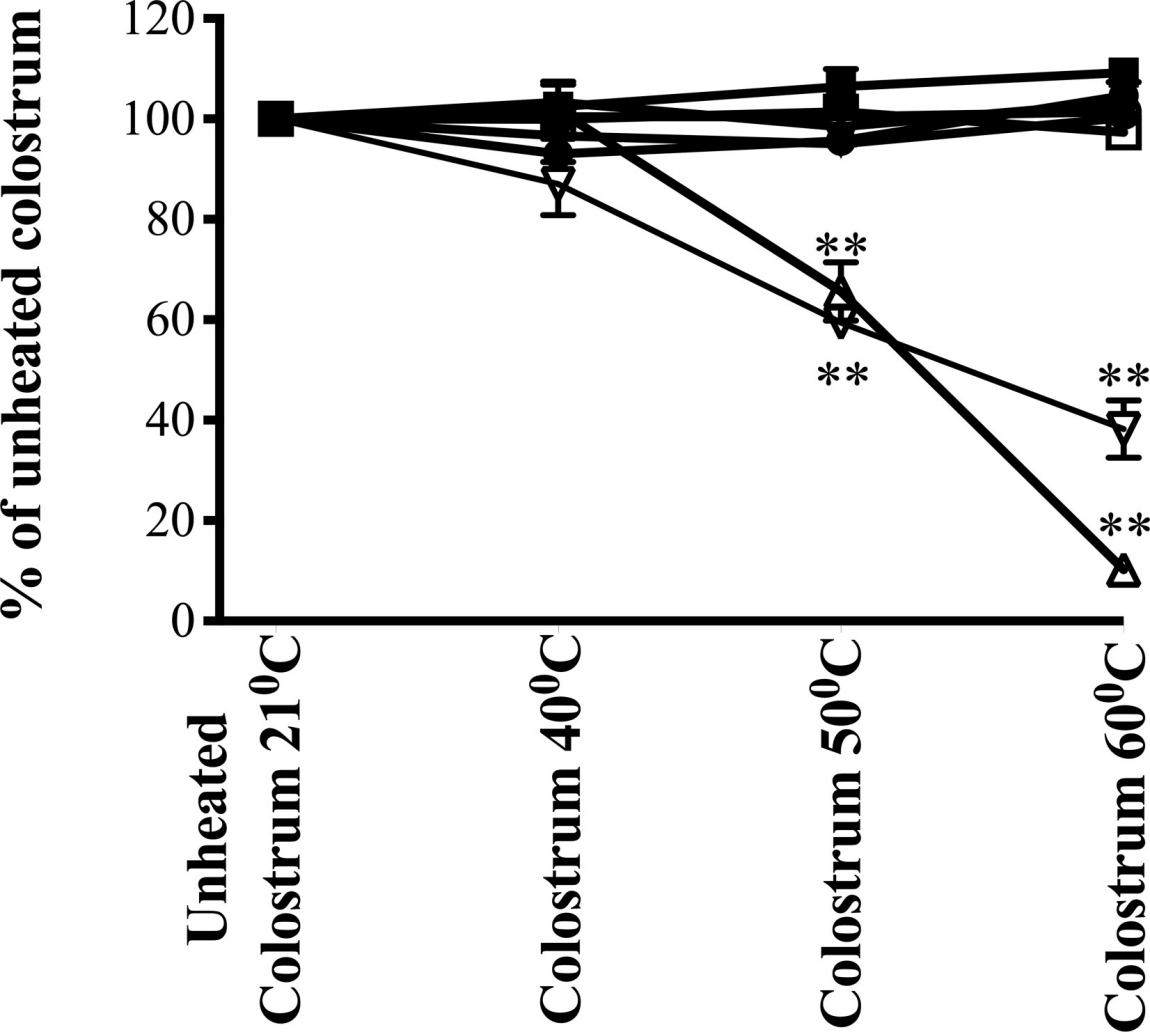
Effect of heating on colostral proliferative and migratory activity and growth factor immunoreactivity. Three 5g samples of sample 9 (from Fig 1A) were heated for 1 h at 40, 50 or 60°C and the fourth kept at laboratory room temperature (21°C, control) throughout. Samples were diluted to 1 mg powder/ml and effects on proliferation (△), cell migration (▽) and growth factor immunoactivity were then determined. In contrast to proliferation and migration results, growth factor immunoreactivity did not change; bovine EGF (▲), bovine TGFβ (▼), bovine haptoglobin (●), bovine betacellulin (◼), and bovine IgG (◯), IGF-1 (☐). Results expressed as % of unheated control, mean +/- SEM of 3 (restitution) or 4 (proliferation and immunoassays) wells per group. ** signifies p< 0.01 vs equivalent bioactivity of unheated (laboratory room temperature) colostrum.

*Heat stability studies*. Subjecting colostrum powder to heat at 50–60°C for 1h caused a progressive fall in bioactivity in both proliferation and migration (Fig 3). In contrast, there was no significant change in bovine EGF, bovine TGFβ, bovine haptoglobin, bovine betacellulin, bovine IGF-1 or bovine IgG immunoreactivity following heating (Fig 3). Total protein concentration of sample was unaffected by dry heating.

## Study series 2

### Variation in bioactivity and growth factor immunoactivity in colostrum samples during days 0–3 after calving

**Fig 4:**
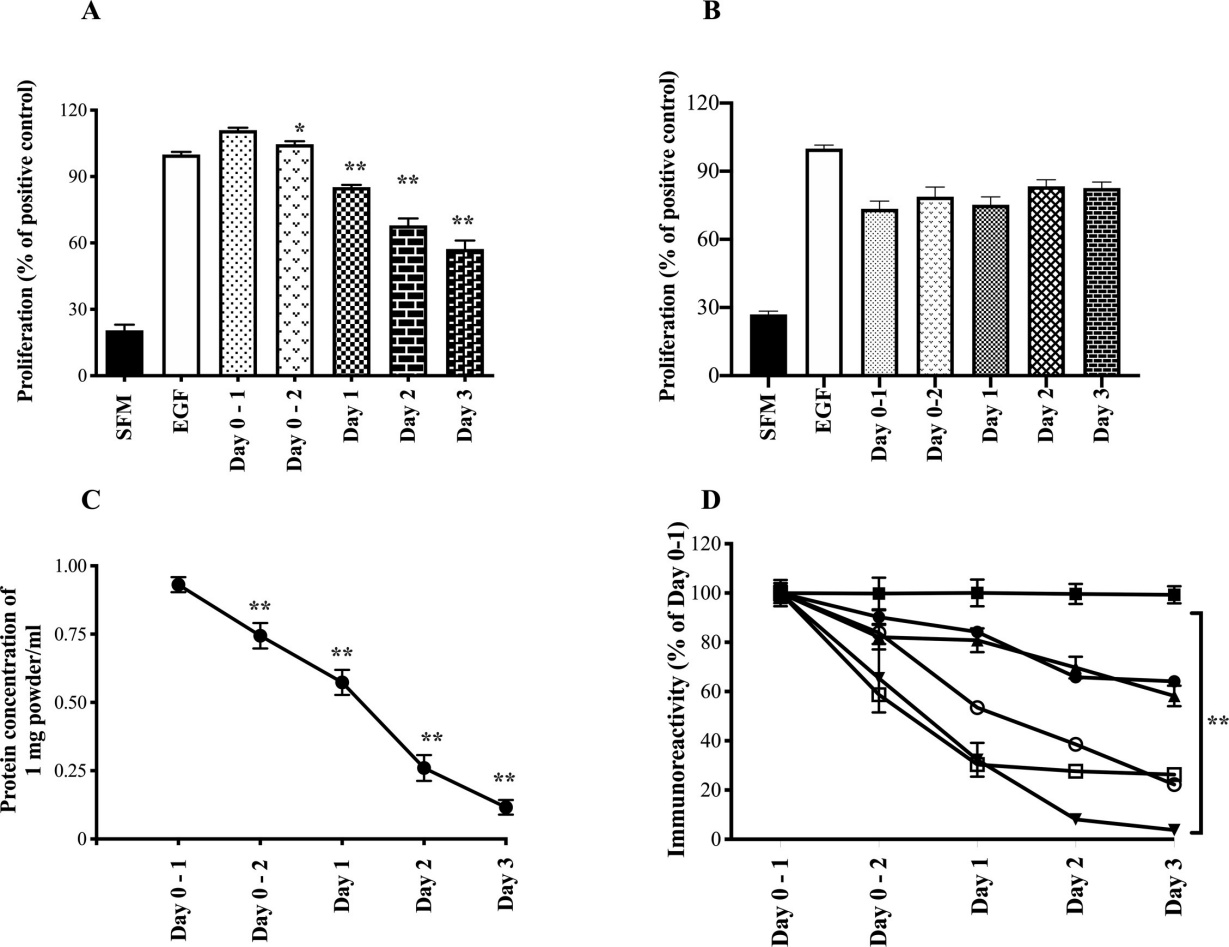
Change in colostral biological and immunoactivity post calving using AGS cells. Colostrum was collected at first and second milking and daily for the following 3 days from 6 cows post calving. Samples were converted into powdered form using freeze drying and subsequently analysed for pro-proliferative activity (AGS cells) using Alamar Blue and growth factor concentrations using commercial ELISA kits. **A)** Proliferative results comparing samples using 1 mg powder/ml. **B)** Proliferative results comparing samples standardised so that each well received 0.4 mg protein/ml. Results expressed as % response compared to effect caused by adding 1μg/ml EGF (positive control, defined as 100%). SFM shows result of serum free medium alone. Results expressed as mean +/- SEM of 6 animals per time point, with each sample measured in quadruplicate. **C**. Change in total protein concentration in the dried colostrum samples over the four-day period. **D**. Growth factor immunoreactivity expressed as % of Day 0–1 sample (absolute values of day 0–1 given in main text); EGF (π), TGFβ (θ), bovine haptoglobin (●), bovine betacellulin (′), IGF-1 (≤) and IgG (○). Results expressed as mean +/- SEM of 6 animals per time point, with each sample measured in triplicate. For A-D, ** signifies p<0.01 vs Day 0–1 value.

When the colostrum samples were compared on a powder weight basis, pro-proliferative activity of colostrum remained high during first and second milkings on day 0. Days two and three showed a progressive drop off in activity such that about two thirds of the activity was lost by day 3 (Fig 4A). Protein concentrations of the samples progressively fell from day 0 to day 3 (Fig 4C). When the same colostrum samples were compared by standardising based on protein content, such that each well received the same amount of total colostrum protein, rather than total weight of the powder, there was no significant difference in pro-proliferative activity across the three days (Fig 4B). Similar results were seen when following pro-migratory activity (S4 Fig).

Immunoassays of growth factors showed the following absolute value results from the initial (Day 0–1) samples; EGF (16.9 +/- 0.7 ng/mg powder), TGFβ (25.7 +/- 4.4 pg/mg powder), haptoglobin (11.1 +/- 0.8 μg/mg powder), betacellulin (405.0 +/- 2.6 ng/mg powder), IGF-1 (438.3 +/- 10.0 ng/mg powder) and IgG (15.36 +/- 0.3 μg/mg powder). To follow changes in levels across all growth factors with very different initial concentrations, results are expressed as percentage change compared to Day 0–1 (Fig 4D). This showed haptoglobin, EGF, TGFβ, IgG, IGF-1 levels paralleled falls in proliferative activity although betacellulin levels remained stable for the test period.

## Discussion

Using a combination of in vitro and in vivo models, we showed marked variability in biological activity of colostrum using assays relevant for gastrointestinal integrity and repair. Some of these differences may relate to the date of colostrum collection following calving, or to transportation or storage conditions of powdered form.

Whatever the initiating injury to the gastrointestinal tract, repair begins within the first few hours by surviving cells at the edge of the wound migrating over the denuded area to re-establish epithelial continuity, a process termed restitution. This is followed by increased proliferation which begins about 24 h following injury and a final remodelling period which occurs over many weeks or months [20]. The proliferation and migration assays used in the current studies have been used by our and other groups previously to compare relative bioactivity of nutritional products (e.g. Ref 16). Consistent results were seen in all three cell lines from stomach, small and large intestinal origin. Six-fold differences in bioactivity were seen when samples were compared on a powder weight basis, as would be taken by the consumer/patient and also when samples were standardised so that the cells received the same amount of total colostrum protein, rather than same amount of powder weight. This excludes the possibility that variation in total colostrum protein content between products was the cause of these differences.

We used a well-established rat indomethacin-restraint gastric damage model to examine whether the differences seen in in vitro bioactivity could have pathophysiological relevance. It has been used by us and others previously to compare relative protective activity between nutritional compounds with therapeutic potential [16]. This model also seemed particularly relevant as colostrum is currently marketed for “*gastric health”*. In keeping with the results from the in vitro studies, the most active colostrum product was three times more effective in reducing gastric injury compared to the worst performing sample from the in vitro study. This finding of concordance between the efficacy in the in vitro proliferation and migration assays and the in vivo model supports the pathophysiological relevance and use of in vitro assays for colostrum quality control and product development purposes.

Indomethacin causes damage to the gastrointestinal tract by several mechanisms including reduction of mucosal prostaglandin levels, reduction of mucosal blood flow, stimulating neutrophil activation, and possibly also stimulating apoptosis [21]. It is likely that many of these mechanisms will be influenced by the numerous growth factors present in the colostrum preparation. The current studies involving Tyrphostin and TGFβ immunoneutralization, in combination with previous experiments involving size exclusion studies of bovine colostrum [22], suggest that EGF, TGFβ_1_ and bovine colostrum derived growth factor are probably all involved in the protective effect. However, the EGFR has multiple ligands [23] and growth factors can act synergistically when added together [24]. In addition, growth factors, such as EGF and TGFα, and cytokines such as IL-1β and IFN-γ stimulate migration through increasing local production of TGFβ_1_ [25]. The inhibitory actions of the TGFβ blocking antibody may, therefore, not solely be due to the neutralisation of TGFβ present within the colostrum.

Following collection, colostrum is usually frozen and stored at the farm and transported in bulk to a processing plant where it is defrosted, pasteurised, and converted to powder form using processes such as spray drying. The end consumer/researcher therefore normally purchases colostrum for personal use or for clinical trials in powdered form. No special provision is given for transportation, and product is delivered via standard freight services with recommended storage conditions usually stated as “store at room temperature”. The current studies demonstrate that even a relatively short exposure of the powdered colostrum to temperature above 40°C has a profound effect on its bioactivity. This finding has direct relevance to the transportation and storage of product for clinical studies being undertaken, especially in hot climates. Importantly, this decrease in bioactivity due to heating was not paralleled by a falloff in immunoreactivity of multiple growth factors present within the colostrum, even at the highest temperature. We therefore consider that bioassays are the optimal method to confirm maintenance of bioactivity for clinical use. Differences in pasteurisation techniques between manufacturers may also be relevant in explaining the variation in bioactivity between commercial colostrum samples as heating to >60°C is performed, however, it is important to note that he temperature sensitivity of bioactivity may be different when colostrum is in its liquid form rather than in its final powdered form. Additional studies would be required to examine this but would be hampered by the fact that the details of the colostrum production (including pasteurisation) are proprietary information and not readily disclosed by the manufacturers.

Our studies examining changes in the milkings immediately following calving showed a rapid drop off in both bioactivity and growth factor concentrations (except for betacellulin) by day 3. These results support previous reports describing similar reductions in total protein, IgG and cytokines such as IL-1β, IL-6 and TNF-α in colostrum over the same period [3, 26]. When the same milkings were assessed for bioactivity using standardised protein content, rather than powder weight, no difference in bioactivity between day zero and day 3 were seen. These results suggest that variation in the proportion of day 0 colostrum used in the commercial products can only be a minor contributor to explaining differences between the commercial products, as differences in bioactivity between the colostrum products remained even when corrected for protein content.

The commercial colostrum samples used for these studies are currently considered food products for marketing legislation purposes. However, as they contain potent biologically active molecules, have been shown to be biologically active in a variety of in vivo models of injury and are being used for medicinal purposes, they should also be considered as *nutraceuticals* (a term combining nutrition and pharmaceutical). Legislation requires tight quality control on conventional medicines where major differences in bioactivity per standard weight of product due to processing or other factors would be considered unacceptable. To interpret results from clinical trials accurately, especially when comparing results from different investigators, the colostrum being used needs to be appropriately quality controlled. Prior to undertaking a clinical trial, we suggest the bioactivity of the colostrum is checked, as relying on weight of powder, protein content, or growth factor immunoassay is ineffective. In addition, researchers need to ensure that the test colostrum product is not exposed to even transient temperature rises above 40°C.

## Supporting information

S1 Fig Effect of colostrum on proliferation: Pilot dose response. AGS cells were incubated in the presence various concentrations of powdered colostrum (0.125–4 mg powder/ml). Cells grown in SFM alone (baseline control) shown as zero concentration of colostrum. Changes in proliferation were assessed by adding Alamar blue and measuring changes in absorbance at 570 nm. Results shown as means +/- SEM of 4 wells per sample and presented as % response compared to effect caused by 1μg/ml EGF (positive control, defined as 100%). ** signifies p<0.01 vs SFM alone.
(TIFF)

S2 Fig Variation in bioactivity of commercial products using RIE-1 cells. RIE-1 cells were incubated in 1 mg powder/ml of 20 different commercial colostrum samples for 24h. Changes in proliferation assessed using (Alamar Blue) (A) and movement of leading edge of wounded monolayers (B) determined. Colostrum sample numbers and colouring remain consistent taken from Fig 1A. Results expressed as % response compared to effect caused by adding 1μg/ml EGF (positive control, defined as 100%). SFM shows result of serum free medium alone. Results expressed as means +/- SEM of 4 wells (proliferation assays) or 3 wells (migration assays). Results of one-way ANOVA showed significant differences between colostrum samples. Labelled means without a common letter are significantly different, P<0.05.
(TIFF)

S3 Fig Variation in bioactivity of commercial products using Caco-2 cells. Caco-2 cells were incubated in 1 mg powder/ml of 20 different commercial colostrum samples for 24h. Changes in proliferation assessed using (Alamar Blue) (A) and movement of leading edge of wounded monolayers (B) determined. Colostrum sample numbers and colouring remain consistent taken from Fig 1A. Results expressed as % response compared to effect caused by adding 1μg/ml EGF (positive control, defined as 100%). SFM shows result of serum free medium alone. Results expressed as means +/- SEM of 4 wells (proliferation assays) or 3 wells (migration assays). Results of one-way ANOVA showed significant differences between colostrum samples. Labelled means without a common letter are significantly different, P<0.05.
(TIFF)

S4 Fig Change in colostral pro-migratory activity post calving using AGS cells. Colostrum was collected at first and second milking and daily for the following 3 days from 6 cows post calving. Samples were then analysed for pro-migratory activity (AGS cells). **A)** Migratory results comparing samples using 1 mg powder/ml. **B)** Migratory results comparing samples standardised so that each well received 0.4 mg protein/ml. Results expressed as % response compared to effect caused by adding 1μg/ml EGF (positive control, defined as 100%). SFM shows result of serum free medium alone. Results expressed as mean +/- SEM of 6 animals per time point, with each sample measured in triplicate. ** signifies p<0.01 vs Day 0–1 value.
(TIFF)

## Abbreviations

EGF: Epidermal growth factor
EGFR: Epidermal growth factor receptor
IGF: insulin-like growth factor
IL: interleukin
NSAID: Nonsteroidal anti-inflammatory drugs
RT: room temperature
SEM: standard error of mean
SFM: serum free medium
TGFβ: Transforming growth factor beta
TNFα: Tumour Necrosis Factor alpha

## References

[1] Playford RJ, Macdonald CE, Johnson WS. Colostrum and milk-derived peptide growth factors for the treatment of gastrointestinal disorders. The American Journal of Clinical Nutrition, 2000;72: 5–14, 10.1093/ajcn/72.1.5 10871554

[2] M'Rabet L, Vos AP, Boehm G, Garssen J. Breast-feeding and its role in early development of the immune system in infants: consequences for health later in life. J Nutr. 2008;138: 1782S–1790S. 10.1093/jn/138.9.1782S 18716187

[3] McGrath BA, Fox PF, McSweeney PLH Kelly AL. Composition and properties of bovine colostrum: a review. Dairy Sci. & Technol. 2016; 96:133–158.

[4] Gauthier SF, Pouliot Y, Maubois J-L. Growth factors from bovine milk and colostrum: composition, extraction and biological activities. Le Lait, INRA Editions, 2006; 86:99–125.

[5] Mihic T, Rainkie D, Wilby KJ, Pawluk SA. The Therapeutic Effects of Camel Milk: A Systematic Review of Animal and Human Trials. J Evid Based Complementary Altern Med. 2016; 21:NP110–126. 10.1177/2156587216658846 27432772

[6] Playford RJ, MacDonald CE, Calnan D, Floyd DN, Podas T, Johnson W, et al Co-administration of the health food supplement, bovine colostrum, reduces the acute non-steroidal anti-inflammatory drug-induced increase in intestinal permeability. Clin Sci (Lond). 2001; 100:627–33. 11352778

[7] Marchbank T, Davison G, Oakes JR, Ghatei MA, Patterson M, Moyer MP et al The nutriceutical bovine colostrum truncates the increase in gut permeability caused by heavy exercise in athletes. Am J Physiol Gastrointest Liver Physiol. 2011;300: G477–G484. 10.1152/ajpgi.00281.2010 21148400

[8] Khan Z, Macdonald C, Wicks AC, Holt MP, Floyd D, Ghosh S, et al Use of the 'nutriceutical', bovine colostrum, for the treatment of distal colitis: results from an initial study. Aliment Pharmacol Ther. 2002;16: 1917–1922. 10.1046/j.1365-2036.2002.01354.x 12390100

[9] Kotsis Y, Mikellidi A, Aresti C, Persia E, Sotiropoulos A, Panagiotakos DB, et al A low-dose, 6-week bovine colostrum supplementation maintains performance and attenuates inflammatory indices following a Loughborough Intermittent Shuttle Test in soccer players. Eur J Nutr. 2018;57: 1181–1195. 10.1007/s00394-017-1401-7 28285432 PMC5861165

[10] Jones AW, March DS, Curtis F, Bridle C. Bovine colostrum supplementation and upper respiratory symptoms during exercise training: a systematic review and meta-analysis of randomised controlled trials. BMC Sports Sci Med Rehabil. 2016; 8:21 10.1186/s13102-016-0047-8 27462401 PMC4960812

[11] Rathe M, Müller K, Sangild PT, Husby S. Clinical applications of bovine colostrum therapy: a systematic review. Nutr Rev. 2014; 72:237–254. 10.1111/nure.12089 24571383

[12] Fogh J, Fogh JM, Orfeo T. One hundred and twenty-seven cultured human tumor cell lines producing tumors in nude mice. J Natl Cancer Inst 1977;59: 221–6. 10.1093/jnci/59.1.221 327080

[13] Barranco SC, Townsend CM Jr, Casartelli C. Establishment and characterization of an in vitro model system for human adenocarcinoma of the stomach. Cancer Res. 1983;43: 1703–1709 6831414

[14] Blay J, Brown KD. Characterization of an epithelioid cell line derived from rat small intestine: demonstration of cytokeratin filaments. Cell Biol Int Rep. 1984;8: 551–60. 10.1016/0309-1651(84)90054-7 6204784

[15] Marchbank T, Weaver G, Nilsen-Hamilton M, Playford RJ. Pancreatic secretory trypsin inhibitor is a major motogenic and protective factor in human breast milk. Am J Physiol Gastrointest Liver Physiol. 2009;296: G697–703. 10.1152/ajpgi.90565.2008 19147803

[16] Playford RJ, Garbowsky M, Marchbank T. Pasteurized Chicken Egg Powder Stimulates Proliferation and Migration of AGS, RIE1, and Caco-2 Cells and Reduces NSAID-Induced Injury in Mice and Colitis in Rats, The Journal of Nutrition, nxaa083, 10.1093/jn/nxaa083 32286629

[17] Marchbank T, Chinery R, Hanby AM, Poulsom R, Elia G, Playford RJ. Distribution and expression of pancreatic secretory trypsin inhibitor and its possible role in epithelial restitution. Am J Pathol. 1996; 14:715‐722. PMC1861739 8774127

[18] Marchbank T, Mahmood A, Fitzgerald AJ, Domin J, Butler M, Goodlad RA, et al Human pancreatic secretory trypsin inhibitor stabilizes intestinal mucosa against noxious agents. Am J Pathol. 2007; 171:1462–1473. 10.2353/ajpath.2007.070192 17982125 PMC2043508

[19] Playford RJ, Vesey DA, Haldane S, Alison MR, Calam J. Dose-dependent effects of fentanyl on indomethacin-induced gastric damage. Digestion 1991;49:198–203. 10.1159/000200722 1797598

[20] Svanes K, Itoh S, Takeuchi K, Silen W. Restitution of the surface epithelium of the in vitro frog gastric mucosa after damage with hypermolar sodium chloride. Gastroenterology 1982;82: 1409–26. 6978275

[21] Levi S, Shaw-Smith C. Non-steroidal anti-inflammatory drugs: how do they damage the gut? Br J Rheumatol 1994;33: 605–12. 10.1093/rheumatology/33.7.605 8019787

[22] Playford RJ, Floyd DN, Macdonald CE, Calnan DP, Adekan RO, Johnson W et al Bovine colostrum is a health food supplement which prevents NSAID induced gut damage. Gut. 1999;44: 653–658. 10.1136/gut.44.5.653 10205201 PMC1727496

[23] Fiske WH, Threadgill D, Coffey RJ. ERBBs in the gastrointestinal tract: recent progress and new perspectives. Exp Cell Res. 2009; 315:583–601. 10.1016/j.yexcr.2008.10.043 19041864 PMC2941795

[24] FitzGerald AJ, Pu M, Marchbank T, Westley BR, May FE, Boyle J, Yadollahi-Farsani M, et al Synergistic effects of systemic trefoil factor family 1 (TFF1) peptide and epidermal growth factor in a rat model of colitis. Peptides. 2004;25: 793–801 10.1016/j.peptides.2003.12.022 15177874

[25] Dignass A, Lynch-Devaney K, Kindon H, Thim L, Podolsky DK. Trefoil peptides promote epithelial migration through a transforming growth factor beta-independent pathway. J Clin Invest. 1994;94: 376–83. 10.1172/JCI117332 8040278 PMC296319

[26] Sobczuk-Szul G, Wielgosz-Groth Z, Wronski M, Rzemiensiewski A. Changes in the bioactive protein concentrations in the bovine colostrum of Jersey and Polish Holstein-Friesian cows. Turk J Vet Anim Sci 2003; 37: 43–49

