# Correction: Marked variability in bioactivity between commercially available bovine colostrum for human use; implications for clinical trials

**DOI:** 10.1371/journal.pone.0240392

**Published:** 2020-10-06

**Authors:** Raymond J. Playford, Meg Cattell, Tania Marchbank

In Table 1, there are errors in the titles of the products tested under “**COLOSTRUM**”, the values under “Total stated protein (g/100 g)” and “Total IgG (g/100g)”, and the “Lot number” values. Please see the correct Table 1 here.

**Table 1:** Commercial colostrum sample information provided by producer.

COLOSTRUM	Total stated protein (g/100g)	Form	Source	Total IgG (g/100g)	Recommended storage	Lot number
Neovite lactose reduced first milk	70	Powder	UK	28	Cool dry place	1805082
Neovite whole colostrum from Welsh farms	55	Powder	UK	16.5	Cool dry place	SA44-01
Neovite cow’s first milk	55	Powder	UK	16.5	Cool dry place	1801007
ColoDan whole colostrum	NS	Powder	Denmark	13	Cool dry place	B5032-017
Bulkpowders colostrum	63.3	Powder	Germany	30	Cool dry place	NS
Biestmilch	70	Capsule	Hawaii	NS	Store for up to 3 year at RT	171241
Vitacost colostrum ultra	NS	Capsule	USA	40	Room Temp 15–30 ^0^C	3823400
Douglas Laboratories colostrum	NS	Powder	New Zealand	NS	Cool dry place 15–25 ^0^C	50153284
Immune Tree colostrum	66.7	Powder	USA	NS	Cool dry place	9902/143
Nutricost	NS	Capsule	USA	NS	NS (shipped at RT)	18010466
NOW colostrum powder	NS	Powder	USA	NS	Cool dry place	3046338
Nutrablast	NS	Capsule	USA	7	Cool dry place	279331
Sovereign Laboratories Colostrum-LD	60	Powder	USA	25	Cool dry place	1802027
Synertek Intact Balanced First colostrum	66.7	Powder	USA	NS	Cool dry place <25 ^0^C	657–30
Renegade Pharmacist	66.7	Powder	USA	22.11	NS (shipped at RT)	9902/221
Sterling Technology colostrum 2070	70	Powder	USA	20	NS (shipped at RT)	0237418
TBR labs peptide ignition colostrum	66.7	Powder	USA	>20	Cool dry place	03028219
Sterling Technology colostrum 3070	70	Powder	USA	30	NS (shipped at RT)	2396–9
Glanbia high fat WPC	88	Powder	USA	NS	NS (shipped at RT)	0068701
Pantheryx Standard colostrum 4515	45	Powder	USA	15	Store at RT for up to 3 years	1141–048203 4518Fi

Table shows source of colostrum samples and product data sheet information for total protein, total IgG, lot numbers and recommended storage conditions, NS = not stated. RT = (store at) room temperature. Storage advice was either present on data sheet or through direct contact with producer. NB list of products are described in random order and do not relate to the order of bioactivity.

Some of the symbols in the caption for Fig 4 are incorrect. Please see the complete, correct Fig 4 caption here. The publisher apologizes for the errors.

**Fig 4:**
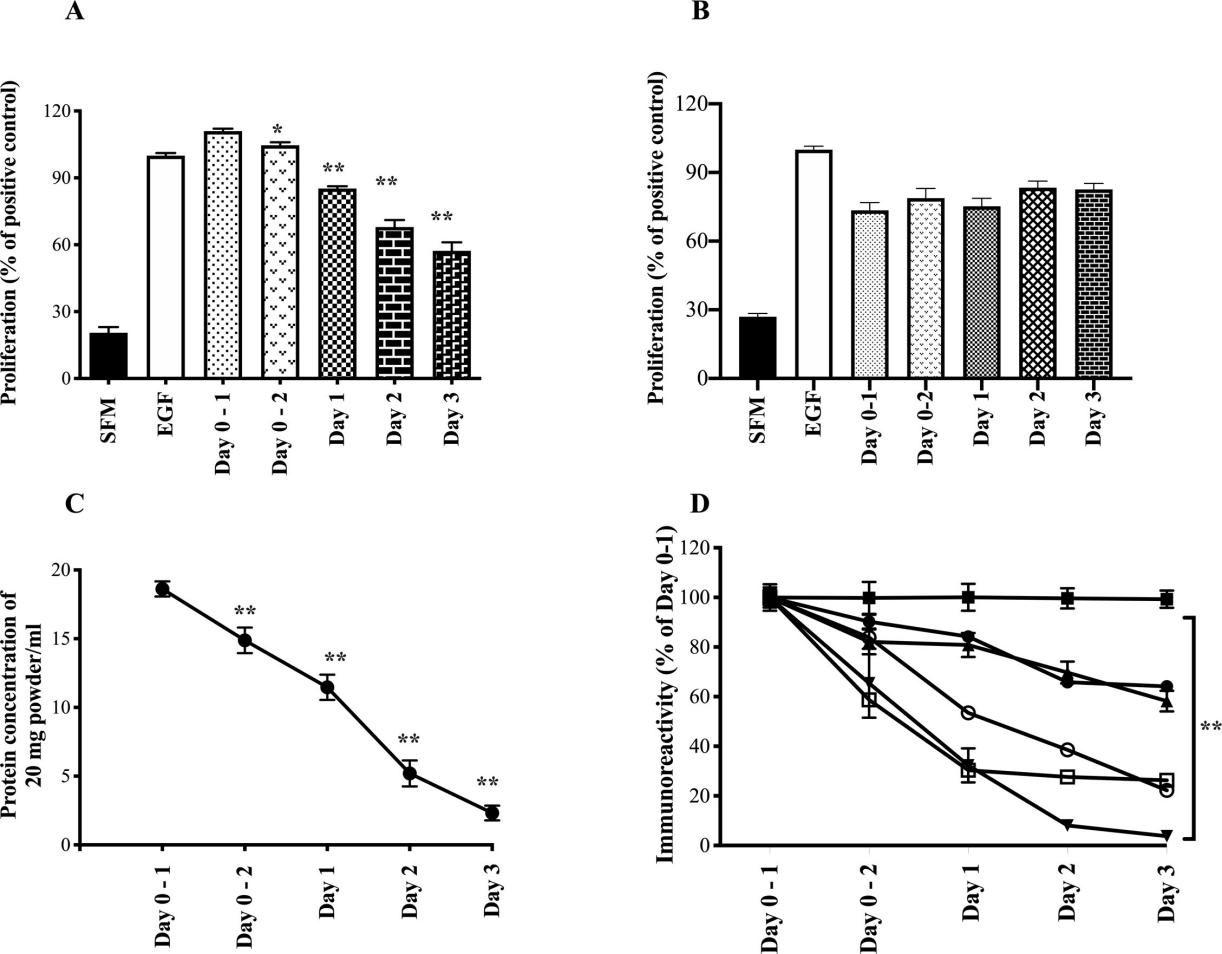
Change in colostral biological and immunoactivity post calving using AGS cells. Colostrum was collected at first and second milking and daily for the following 3 days from 6 cows post calving. Samples were then analysed for pro-proliferative activity (AGS cells) using Alamar Blue and growth factor concentrations using commercial ELISA kits. A. Proliferative results comparing samples using 1 mg powder/ml. B. Proliferative results comparing samples standardised so that each well received 0.4 mg protein/ml. Results expressed as % response compared to effect caused by adding 1μg/ml EGF (positive control, defined as 100%). SFM shows result of serum free medium alone. Results expressed as mean +/- SEM of 6 animals per time point, with each sample measured in quadruplicate. C. Change in total protein concentration in the dried colostrum samples over the four-day period. D. Growth factor immunoreactivity expressed as % of Day 0–1 sample (absolute values of day 0–1 given in main text); EGF (▲), TGFβ (▼), bovine haptoglobin (●), bovine betacellulin (■), IGF-1 (□) and IgG (○). Results expressed as mean +/- SEM of 6 animals per time point, with each sample measured in triplicate. For A-D, ** signifies p<0.01 vs Day 0–1 value.
