# Supplementary figures and images for: Marked variability in bioactivity between commercially available bovine colostrum for human use; implications for clinical trials
Source: PLoS One. 2020 Jun 17;15(6):e0234719. doi: 10.1371/journal.pone.0234719 (PMC7299325; doi:10.1371/journal.pone.0234719)

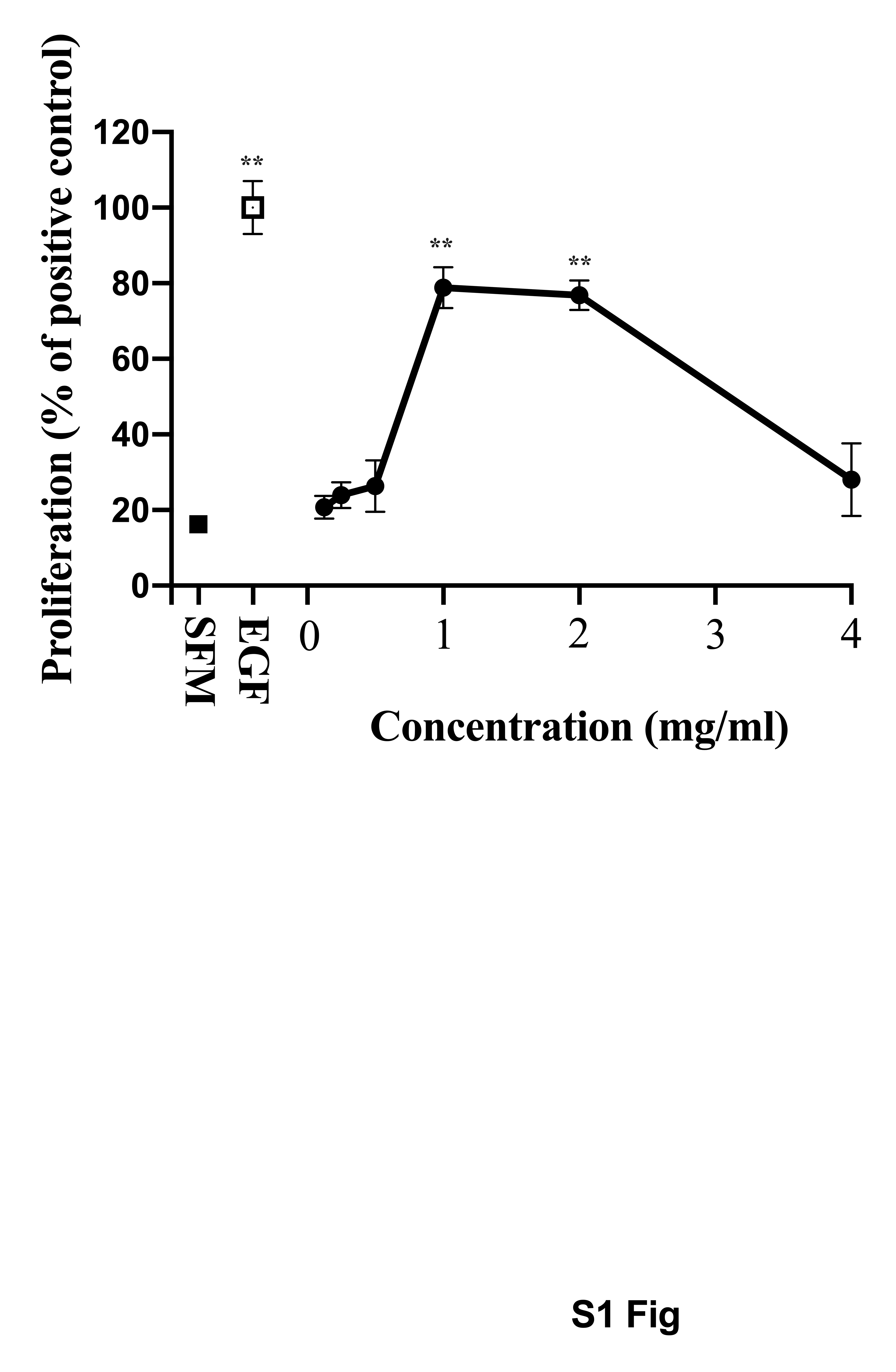

Supplement: S1 Fig — AGS cells were incubated in the presence various concentrations of powdered colostrum (0.125–4 mg powder/ml). Cells grown in SFM alone (baseline control) shown as zero concentration of colostrum. Changes in proliferation were assessed by adding Alamar blue and measuring changes in absorbance at 570 nm. Results shown as means +/- SEM of 4 wells per sample and presented as % response compared to effect caused by 1μg/ml EGF (positive control, defined as 100%). ** signifies p<0.01 vs SFM alone. (TIFF) [file pone.0234719.s001.tiff]

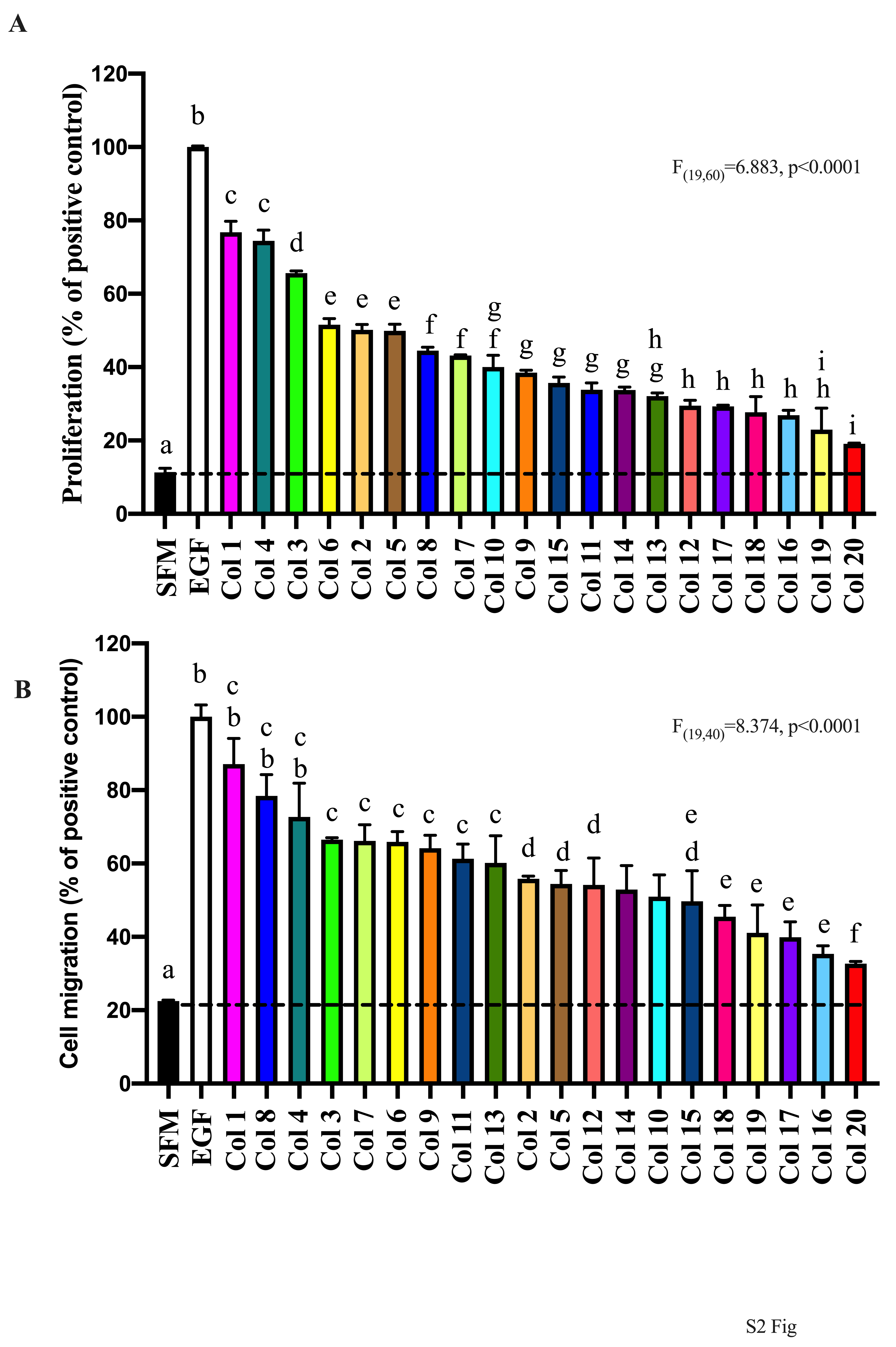

Supplement: S2 Fig — RIE-1 cells were incubated in 1 mg powder/ml of 20 different commercial colostrum samples for 24h. Changes in proliferation assessed using (Alamar Blue) (A) and movement of leading edge of wounded monolayers (B) determined. Colostrum sample numbers and colouring remain consistent taken from Fig 1A. Results expressed as % response compared to effect caused by adding 1μg/ml EGF (positive control, defined as 100%). SFM shows result of serum free medium alone. Results expressed as means +/- SEM of 4 wells (proliferation assays) or 3 wells (migration assays). Results of one-way ANOVA showed significant differences between colostrum samples. Labelled means without a common letter are significantly different, P<0.05. (TIFF) [file pone.0234719.s002.tiff]

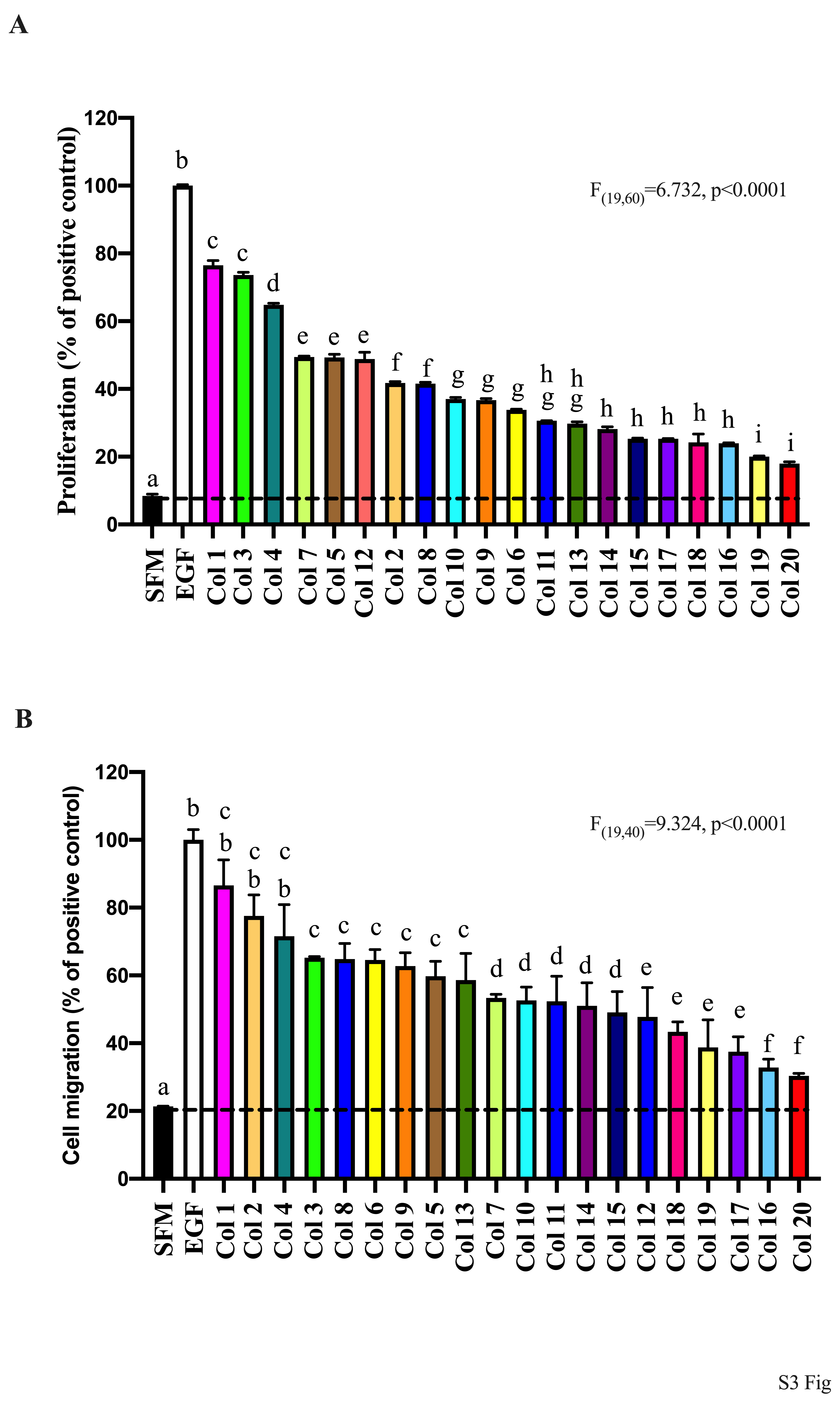

Supplement: S3 Fig — Caco-2 cells were incubated in 1 mg powder/ml of 20 different commercial colostrum samples for 24h. Changes in proliferation assessed using (Alamar Blue) (A) and movement of leading edge of wounded monolayers (B) determined. Colostrum sample numbers and colouring remain consistent taken from Fig 1A. Results expressed as % response compared to effect caused by adding 1μg/ml EGF (positive control, defined as 100%). SFM shows result of serum free medium alone. Results expressed as means +/- SEM of 4 wells (proliferation assays) or 3 wells (migration assays). Results of one-way ANOVA showed significant differences between colostrum samples. Labelled means without a common letter are significantly different, P<0.05. (TIFF) [file pone.0234719.s003.tiff]

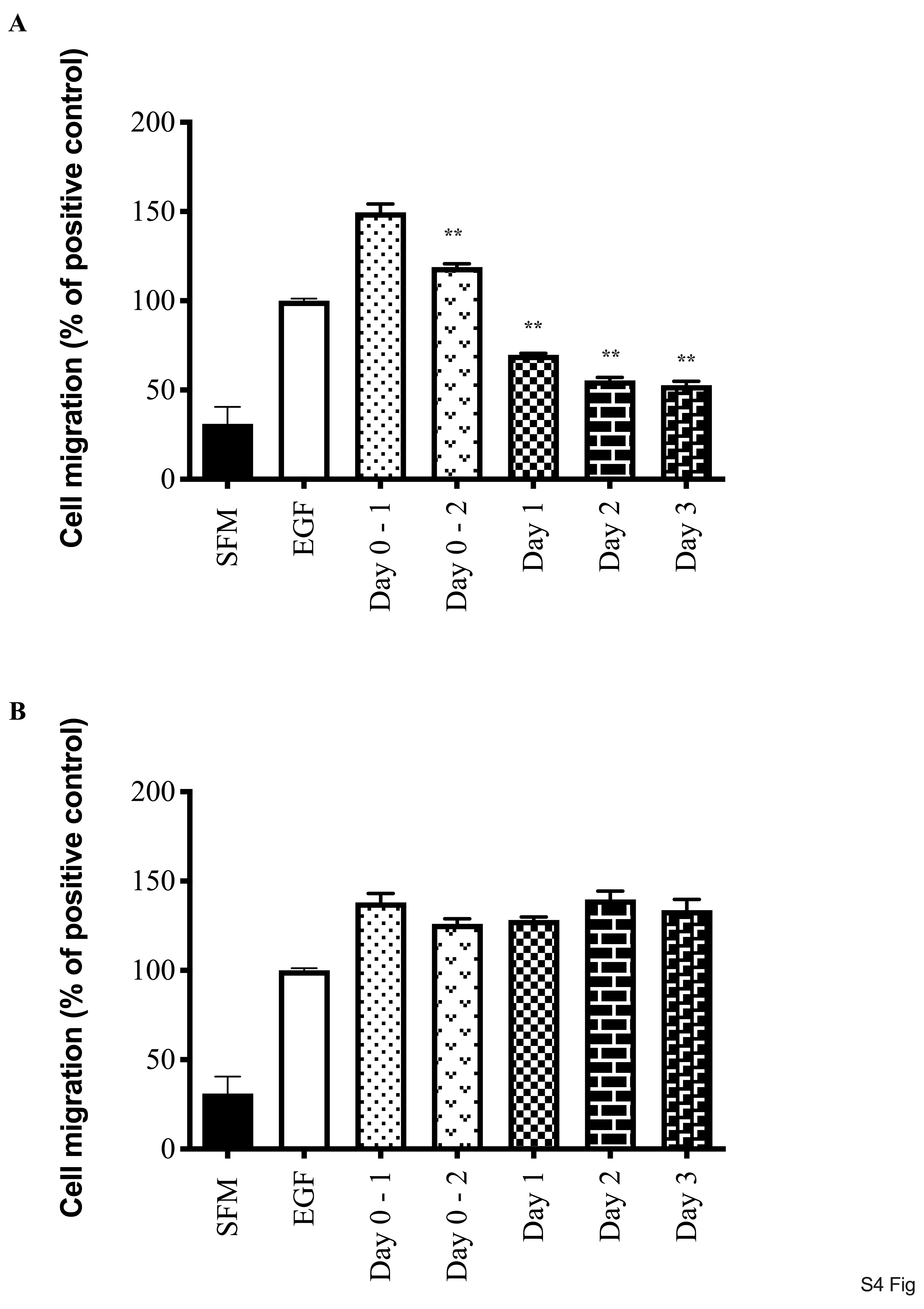

Supplement: S4 Fig — Colostrum was collected at first and second milking and daily for the following 3 days from 6 cows post calving. Samples were then analysed for pro-migratory activity (AGS cells). A) Migratory results comparing samples using 1 mg powder/ml. B) Migratory results comparing samples standardised so that each well received 0.4 mg protein/ml. Results expressed as % response compared to effect caused by adding 1μg/ml EGF (positive control, defined as 100%). SFM shows result of serum free medium alone. Results expressed as mean +/- SEM of 6 animals per time point, with each sample measured in triplicate. ** signifies p<0.01 vs Day 0–1 value. (TIFF) [file pone.0234719.s004.tiff]
